# Exploring the efficacy of memory specificity training on depression among Iranian adolescents: a comparative analysis of online vs. in-person delivery

**DOI:** 10.1038/s41598-024-68709-9

**Published:** 2024-09-28

**Authors:** Mohsen Salamat, Alireza Moradi, Jafar Hasani, Sharareh Farahimanesh, Fateme Ayatmehr, Hanieh Yavarzadeh, Laura Jobson

**Affiliations:** 1 Department of Clinical Psychology, Kharazmi University, Tehran, Iran; 2 Department of Clinical Psychology, Kharazmi University and Institute for Cognitive Sciences Studies, Tehran, Iran; 3 Institute for Cognitive and Brain Sciences, Shahid Beheshti University, Tehran, Iran; 4 School of Psychological Sciences, Monash University, Melbourne, 3800 Australia

**Keywords:** Memory specificity training, Computerized memory specificity training, Depression, Adolescents, Emotion regulation, Cognitive control, Psychology, Health care

## Abstract

Depression in adolescence is common worldwide, with the burden being highest in low- and middle-income countries. This study assessed the efficacy of in-person Memory Specificity Training (MeST) and computerized MeST (c-MeST) as cognitive training programs aimed at addressing depression among Iranian adolescents. A secondary aim was to evaluate the efficacy of MeST and c-MeST on autobiographical memory specificity, emotion regulation and cognitive control. Ninety Iranian male adolescents (aged 13–18 years) with depression were randomly assigned to three groups; MeST group (*n* = 30), c-MeST group (*n* = 30) and the non-active control group (*n* = 30). Participants completed the Beck Depression Inventory-II, Autobiographical Memory Test, Cognitive Emotion Regulation Questionnaire, Wisconsin Card Sorting Test and Stroop Color and Word Test. The groups underwent either MeST and c-MeST. All the assessments were re-conducted after the intervention (post-intervention) and at 1-month post-intervention (follow-up). The in-person MeST group exhibited significantly higher autobiographical memory specificity at post-intervention and follow-up compared to the c-MeST group. Both groups demonstrated significantly lower levels of depression at post-intervention and follow-up. Both groups showed improvements in emotion regulation and cognitive control, which were found to mediate improvements in depression symptomatology. c-MeST and MeST appear promising brief interventions for the treatment of depression among adolescents in Iran.

## Introduction

Depression is a widespread mental health condition, impacting around 8% of youth globally^1^. Adolescence is a stage characterized by an increased susceptibility to the emergence of depression^2^. Adolescent depression manifests through heightened social isolation and loneliness, accompanied by reduced school attendance and performance^3,4^. Persistent depression during adolescence can extend into adulthood, heightening the risks of relapse, suicidal tendencies, and amplified functional impairments in both social and academic environments^5,6^. Recognizing cognitive challenges among youth with depression provides valuable insights for shaping effective treatment strategies, which may contribute to mitigating the risk of relapse and minimizing adverse long-term consequences^5^. While considerable research has focused on the treatment of depression among adolescents, much of this research has been conducted within high-income countries, despite the burden of depression among adolescents being highest in low- and middle-income countries^7^. Thus, there is a need to further investigate cognitive factors that can be targeted in treatments for adolescents with depression in regions such as Iran, where depression and suicide rates among adolescents is a source of increasing concern^8–10^. In Iran around one third of school students (32% among middle-school students and 47% among high-school students) experience some degree of depression, and the prevalence of depression is twice as high in girls compared to boys^11^. The Vice President of Iran’s Suicide Prevention Scientific Society recently noted concerns regarding suicide attempts and deaths among youth in Iran^12^, with the rate of death by suicide among boys tending to be higher^13^. This gender paradox (i.e., suicide rate being higher among boys, despite depression rates being higher among girls), may reflect suicide rates being higher among boys whilst suicide attempts being higher among girls^14^. Additionally, gender differences in suicidal behaviour may reflect differences in behavioral and emotional problems, with higher rates of suicide deaths among male youths being associated with a higher prevalence of externalizing disorders, while female youth are more prone to internalizing disorders^14^.

A consistent cognitive predictor of the development and maintenance of depression involves challenges in recalling specific past events^15^. Individuals experiencing depression, in comparison to those without depression, frequently exhibit difficulty recalling memories of specific events that occurred within a single day. Instead, they tend to offer memories of experiences spanning longer durations, categories of repeated events, or abstract representations of various experiences^15^. This cognitive challenge, known as overgeneral memory (OGM), is a contributing factor to the development and persistence of clinical depression^16^, including among adolescents^17^. Importantly, research has demonstrated that deficits in autobiographical memory specificity can be modified, and targeting autobiographical memory specificity can lead to improvements in depression^18^, including among adolescents^19^.

Memory Specificity Training (MeST) is an evidence-based low-intensity intervention targeting autobiographical memory specificity^18^. It involves participants practicing providing specific (detailed and elaborated) memories in response to positive, negative and neutral valanced cue words^20^. MeST has been found to lead to significant improvements in the retrieval of specific memories and reductions in depression^17–20^. More recently, computerized MeST (C-MeST), an on-line, low-cost, low-resource, easily accessible intervention, has been developed^16,21,22^. Given the low resource and low-intensity focus of MeST, particularly c-MeST, these psychological interventions may be of particular relevance in Iran.

Emotion regulation and cognitive control play central roles in the inability to provide specific memories in depression^15,18,23^. Emotion regulation (i.e., the ability to utilize strategies to enhance, sustain, or diminish the intensity, duration, and course of emotions^24^) relates to the presence of OGM and can account for the association between overgenerality and psychopathology^25^. It has been proposed that an OGM retrieval style may be a form of emotion regulation, as general memories are thought to result in less affect than specific memories, and maladaptive emotion regulation may use up cognitive capacity needed to search and retrieve a specific memory^23^. Acquiring the ability to manage emotions is a crucial socio-emotional skill that enables adaptability in emotionally charged situations^26^. Considering the heightened independence and new challenges in adolescence, adolescents might have specific requirements to manage emotions when faced with stressors. Failing to do so may increase the risk of mental health issues. Therefore, emotion regulation may be a crucial element in understanding the intricate factors contributing to depression^27^. As the role of OGM in depression may, in part, be due to the functions of specific autobiographical memories in emotion regulation^18^, MeST and c-MeST may not only improve autobiographical memory specificity but also emotion regulation in the treatment of depression. It is essential to thoroughly examine the developmental facets of emotion regulation in adolescence when identifying focal points for preventing and intervening early in mental health concerns^26^.

OGM is also associated with impairments in cognitive control, which may impact therapeutic outcomes^15,18,23^. Cognitive control involves the ability to focus on and respond to information that aligns with goals, while suppressing attention and responses to distracting, irrelevant inputs^28^. It encompasses various executive control processes, including updating, switching, and inhibition^29^. Deficiencies in cognitive control may impede one’s ability to successfully search for and retrieve specific memories, as difficulties in inhibiting irrelevant information and switching between and holding information may disrupt the search for a specific memory^23^. Thus, it is important to examine whether MeST and c-MeST have the potential to improve factors, such as emotion regulation and cognitive control, known to be associated with OGM and play a role in the maintenance of depression among adolescents.

### Current study

The primary objective of this study was to compare the efficacy of in-person MeST and c-MeST as cognitive training programs targeting impaired autobiographical memory specificity among adolescents with depression in Iran. Specifically, our first aim was to examine whether MeST and c-MEST could reduce symptoms of depression at post-intervention and 1-month follow-up, when compared to a non-active control group (i.e., a group that did not undergo any tasks or interventions). Our secondary aims were to explore whether MeST and c-MEST could improve memory specificity, emotion regulation and cognitive control at post-intervention and 1-month follow-up, when compared to a non-active control group. Finally, following the approach of Neshat-Doost and colleagues^19^, we explored whether any changes in memory specificity, emotion regulation and cognitive control over the course of training mediated depression symptom improvement at follow-up. This is important to consider as it relates to the theoretically predicted causal relationships between these variables and improvements in depression symptomatology^19,23^.

## Results

**Figure 1:**
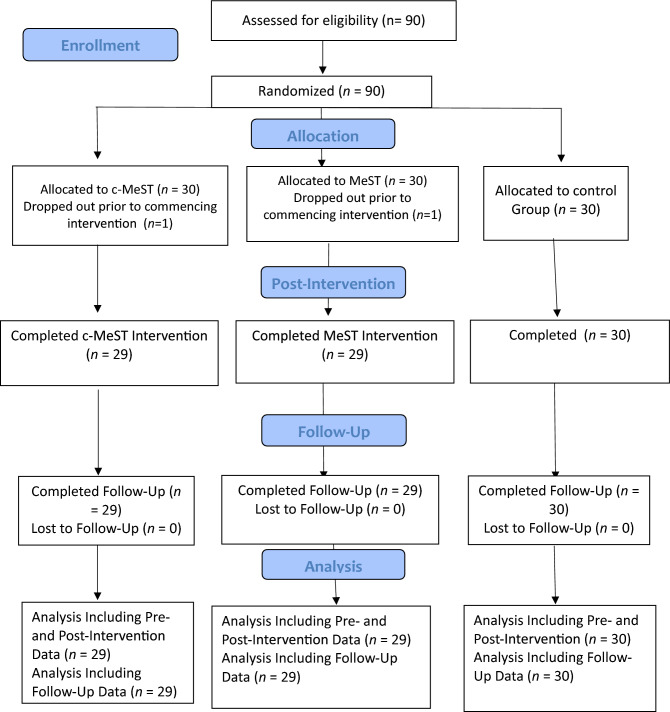
CONSORT (consolidated standards of reporting trials) diagram of the progress through the phases of the randomized trial.

Table 1 presents details of the three groups and highlights the three groups did not differ significantly at baseline. Two participants (MeST *n* = 1; c-MeST *n* = 1) dropped out of the study following randomization (i.e., prior to commencing the interventions). Those who commenced the interventions attended and completed every intervention and assessment session. Overall, participants completed the homework tasks as instructed. The PRISMA diagram is presented in Fig. 1 and the CONSORT checklist was followed and is available by contacting the authors.

**Table 1:** Descriptive statistics for MeST, c-MeST and control groups.

Variable		c-MeST group		MeST group		Control group	
		*M*	*SD*	*M*	*SD*	*M*	*SD*
Memory specificity	Baseline	2.90	2.68	1.69	1.93	2.43	2.69
	Post-Intervention	5.66	2.54	6.97	2.46	2.43	2.87
	Follow-up	7.86	2.39	9.97	1.87	2.50	3.05
Adaptive emotion regulation	Baseline	23.52	2.65	21.66	4.15	25.93	6.01
	Post-Intervention	40.79	2.34	39.90	3.83	24.17	6.16
	Follow-up	44.07	2.31	44.86	2.61	22.27	5.64
Maladaptive emotion regulation	Baseline	29.17	3.62	26.41	3.74	20.00	6.16
	Post-Intervention	13.62	2.31	13.90	3.38	20.67	6.36
	Follow-up	10.93	1.60	10.72	2.14	23.77	5.21
Cognitive control							
SCWT-interference error	Baseline	4.27	2.58	5.55	1.88	4.00	3.33
	Post-intervention	1.06	1.41	1.72	1.41	4.43	2.96
	Follow-up	0.44	0.86	0.51	0.73	4.46	1.88
SCWT-interference time	Baseline	25.99	12.51	28.46	9.68	27.82	8.19
	Post-intervention	20.56	9.99	22.96	7.24	19.54	7.50
	Follow-up	17.60	8.92	17.59	6.24	17.62	7.68
WCRT-perseverative errors	Baseline	21.90	4.07	27.69	3.58	27.93	2.70
	Post-Intervention	5.93	2.02	4.62	2.37	22.73	7.22
	Follow-up	4.21	1.35	2.69	0.85	16.67	5.58
WCRT-non-perseverative errors	Baseline	28.90	4.84	28.21	3.71	27.43	3.84
	Post-Intervention	9.45	3.92	5.10	2.54	21.83	6.09
	Follow-up	2.03	0.82	1.45	0.83	16.53	5.22

*CERQ* Cognitive Emotion Regulation Questionnaire, *SCWT* Stroop Color and Word Test, *WCRT* Wisconsin Card Sorting Test.

### Objective 1: depression symptoms

**Figure 2:**
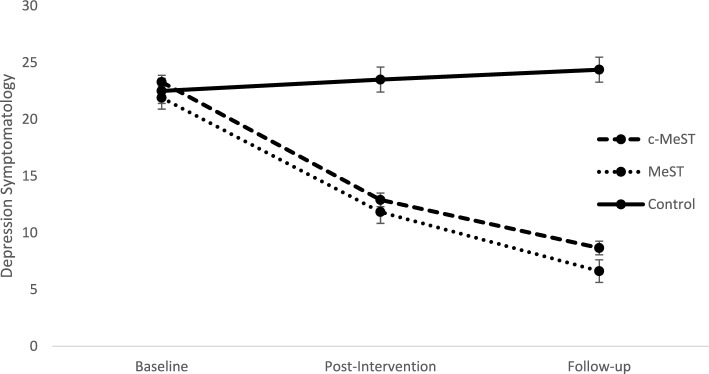
Mean Depression Symptomatology for the MeSt Group, c-MeSt Group and Control Group at Baseline, Post-Intervention and Follow-Up.

As shown in Fig. 2, the group × time interaction was significant for depression, *F* (2.70, 115.50) = 115.23, *p* < 0.05, *η*^2^ = 0.73. Post-hoc analyses demonstrated the c-MeST group had significantly less depression symptomatology than Controls at post-intervention; *t*(57) = 8.12, *p* < 0.001, *d* = 2.12, and follow-up, *t*(57) = 11.13, *p* < 0.001, *d* = 2.94. Similarly, the MeST group had significantly less depression symptomatology than Controls at post-intervention; *t*(57) = 9.02, *p* < 0.001, *d* = 2.36, and follow-up, *t*(57) = 13.47, *p* < 0.001, *d* = 3.57. While the c-MeST group and MeST groups did not differ significantly at post-intervention; *t*(56) = 1.05, ns, *d* = 0.29, the c-MeST group had significantly greater depression symptomatology than the MeST group at follow-up; *t*(56) = 2.66, *p* < 0.05, *d* = 1.22. Within-group analyses showed both the MeST and c-MeST groups had significantly lower levels of depression at post-intervention and follow-up, when compared to baseline, MeST group: post-intervention, *t*(28) = 26.65, *p* < 0.001, *d* = 2.49, follow-up, *t*(28) = 18.70, *p* < 0.001, *d* = 4.53; c-MeST group: post-intervention, *t*(28) = 20.66, *p* < 0.001, *d* = 2.20, follow-up, *t*(28) = 17.02, *p* < 0.001, *d* = 3.20. The Control group showed no significant changes at post-intervention, *t*(29) = 1.74, ns, *d* = 0.17, or follow-up, *t*(29) = 1.68, ns, *d* = 0.13.

### Secondary objectives

The group means for the secondary objectives are presented in Table 1.

#### Autobiographical memory

The group × time interaction was significant for autobiographical memory specificity, *F* (2.87, 122.18) = 43.38, *p* < 0.05, *η*^2^ = 0.50. As shown in Table 2, post-hoc analyses found both the c-MeST and MeST groups recalled significantly more specific memories at post-intervention and follow-up than the Control group. The MeST group provided more specific memories that the c-MeST group at post-intervention and follow-up. While the MeST and c-MEST groups provided significantly more specific memories at post-intervention and follow-up, the control group showed no significant change.

**Table 2:** Summary of follow-up analyses for the secondary outcomes.

Variable	Group comparison	Statistics	Summary
Autobiographical memory specificity			
Post-intervention			
Between Group	c-MeST vs Control	*t*(57) = 4.55, *p* < 0.001, *d* = 1.19	c-MeST > Control
	MeST vs Control	*t* (57) = 6.50, *p* < 0.001, *d* = 1.70	MeST > Control
	c-MeST vs MeST^a^	*t* (56) = 1.99, *p* = 0.05, *d* = 0.53	MeST > c-MeST
Within Group	c-MeST	*t* (28) = 5.77, *p* < 0.001, *d* = 1.06	Improvement
	MeST	*t* (28) = 10.14, *p* < 0.001, *d* = 2.39	Improvement
	Control	*t* (29) < 0.001, *ns*	No change
Follow-up			
Between Group	c-MeST vs Control	*t* (57) = 7.50, *p* < 0.001, *d* = 1.96	c-MeST > Control
	MeST vs Control	*t* (57) = 9.93, *p* < 0.001, *d* = 2.60	MeST > Control
	c-MeST vs MeST^a^	*t* (56) = 2.14, *p* < 0.05, *d* = 0.57	MeST > c-MeST
Within Group	c-MeST	*t* (28) = 9.15,* p* < 0.001, *d* = 1.96	Improvement
	MeST	*t* (28) = 15.14, *p* < 0.001, *d* = 3.89	Improvement
	Control	*t* (29) = 2.84, *ns*	No change
Adaptive emotion regulation			
Post-intervention			
Between Group	c-MeST vs Control	*t* (57) = 13.61, *p* < 0.001, *d* = 3.57	c-MeST > Control
	MeST vs Control	*t* (57) = 11.73*, p* < 0.001, *d* = 3.07	MeST > Control
	c-MeST vs MeST	*t* (56) = 0.36, *ns*	c-MeST = MeST
Within Group	c-MeST	*t* (28) = 37.55*, p* < 0.001*, d* = 6.92	Improvement
	MeST	*t* (28) = 26.76, *p* < 0.001,* d* = 4.57	Improvement
	Control	*t* (29) = 3.30, *ns*	No change
Follow-up			
Between Group	c-MeST vs Control	*t* (57) = 19.29*, p* < 0.001*, d* = 5.06	c-MeST > Control
	MeST vs Control	*t* (57) = 19.61*, p* < 0.001, *d* = 5.14	MeST > Control
	c-MeST vs MeST	*t* (56) = 1.22, *ns*	MeST > c-MeST
Within Group	c-MeST	*t* (28) = 7.57, *p* < 0.001, d = 1.40	Improvement
	MeST	*t* (29) = 9.19, *p* < 0.001, d = 1.51	Improvement
	Control	*t* (29) = 3.08*, ns*	No change
Maladaptive emotion regulation			
Post-intervention			
Between Group	c-MeST vs Control	*t* (57) = 5.61*, p* < 0.001, *d* = 1.47	c-MeST < Control
	MeST vs Control	*t* (57) = 5.07*, p* < 0.001, *d* = 1.33	MeST < Control
	c-MeST vs MeST	*t* (56) = 0.36, *ns*	c-MeST = MeST
Within Group	c-MeST	*t* (28) = 24.80, p < 0.001*, d* = 5.13	Improvement
	MeST	*t* (28) = 20.97, *p* < 0.001, *d* = 3.51	Improvement
	Control	*t *(29) = 1.47, *ns*	No change
Follow-up			
Between Group	c-MeST vs Control	*t* (57) = 12.69*, p* < 0.001,* d* = 3.33	c-MeST < Control
	MeST vs Control	*t* (57) = 12.49*, p* < 0.001*, d* = 3.28	MeST < Control
	c-MeST vs MeST	*t* (56) = 0.41*, ns*	MeST = c-MeST
Within Group	c-MeST	*t* (28) = 6.25*, p* < 0.001, *d* = 1.35	Improvement
	MeST	*t* (29) = 7.98, *p* < 0.001, *d* = 1.12	Improvement
	Control	*t* (29) = 5.45*, p* < 0.001,* d* = 0.53	Improvement
Cognitive control			
Perseverative errors			
Post-intervention			
Between Group	c-MeST vs Control	*t* (57) = 12.01*, p* < 0.001*, d* = 3.17	c-MeST < Control
	MeST vs Control	*t* (57) = 12.85, *p* < 0.001, *d* = 2.27	MeST < Control
	c-MeST vs MeST^a^	*t* (56) = 2.26, *p* < 0.05, *d* = 0.60	c-MeST > MeST
Within Group	c-MeST	*t* (28) = 26.12,* p* < 0.001*, d* = 5.93	Improvement
	MeST	*t* (28) = 34.03, *p* < 0.001, *d* = 7.50	Improvement
	Control	*t* (29) = 4.23*, p* < 0.001*, d* = 0.95	Improvement
Follow-up			
Between Group	c-MeST vs Control	*t* (57) = 11.69*, p* < 0.001, *d* = 3.08	c-MeST < Control
	MeST vs Control	*t* (57) = 13.33, *p* < 0.001, *d* = 3.51	MeST < Control
	c-MeST vs MeST^a^	*t* (56) = 5.13, *p* < 0.05, *d* = 1.35	c-MeST > MeST
Within Group	c-MeST	*t* (28) = 3.86,* p* < 0.001*, d* = 1.13	Improvement
	MeST	*t* (28) = 4.42, *p* < 0.001, *d* = 1.14	Improvement
	Control	*t* (29) = 11.50,* p* < 0.001, *d* = 0.93	Improvement
Non-perseverative errors			
Post-intervention			
Between Group	c-MeST vs Control	*t* (57) = 9.25,* p* < 0.001, *d* = 2.42	c-MeST < Control
	MeST vs Control	*t* (57) = 13.69, *p* < 0.001, *d* = 3.59	MeST < Control
	c-MeST vs MeST^a^	*t* (56) = 5.01, *p* < 0.05, *d* = 1.32	c-MeST > MeST
Within Group	c-MeST	*t* (28) = 30.36,* p* < 0.001*, d* = 4.42	Improvement
	MeST	*t* (28) = 32.40, *p* < 0.001, *d* = 7.28	Improvement
	Control	*t* (29) = 5.37,* p* < 0.001,* d* = 1.10	Improvement
Follow-up			
Between Group	c-MeST vs Control	*t* (57) = 14.78,* p* < 0.001*, d* = 3.89	c-MeST < Control
	MeST vs Control	*t* (57) = 15.37, *p* < 0.001, *d* = 4.04	MeST < Control
	c-MeST vs MeST^a^	*t* (56) = 2.70, *p* < 0.05, *d* = 0.71	MeST < c-MeST
Within Group	c-MeST	*t* (28) = 10.52, *p* < 0.001, *d* = 2.62	Improvement
	MeST	*t* (28) = 7.75, *p* < 0.001, *d* = 1.93	Improvement
	Control	*t* (29) = 9.76, *p* < 0.001, *d* = 0.93	Improvement
Stroop—errors			
Post-intervention			
Between Group	c-MeST vs Control	*t* (57) = 5.52, *p* < 0.001, *d* = 1.45	c-MeST < Control
	MeST vs Control	*t* (57) = 4.45, *p* < 0.001, *d* = 1.17	MeST < Control
	c-MeST vs MeST	*t* (56) = 1.76, *ns*	c-MeST = MeST
Within Group	c-MeST	*t* (28) = 7.29, *p* < 0.001, *d* = 1.54	Improvement
	MeST	*t* (28) = 11.89, *p* < 0.001, *d* = 2.30	Improvement
	Control	*t* (29) = 1.65, *ns*	No change
Follow-up			
Between Group	c-MeST vs Control	*t* (57) = 10.43, *p* < 0.001, *d* = 2.75	c-MeST < Control
	MeST vs Control	*t* (57) = 10.50, *p* < 0.001, *d* = 2.77	MeST < Control
	c-MeST vs MeST	*t* (56) = 0.32, *p ns*	c-MeST = MeST
Within Group	c-MeST	*t* (28) = 2.76, *p* = 0.01, *d* = 0.53	Improvement
	MeST	*t* (28) = 4.82, *p* < 0.001, *d* = 1.07	Improvement
	Control	*t* (29) = 1.01, *ns*	Improvement
Stroop—interference time			
Post-intervention			
Between Group	c-MeST vs Control	*t* (57) = 0.48, *ns*	c-MeST = Control
	MeST vs Control	*t* (57) = 1.77, ns	MeST = Control
	c-MeST vs MeST	*t* (56) = 1.00, *ns*	c-MeST = MeST
Within Group	c-MeST	*t* (28) = 6.55, *p* < 0.001, *d* = 0.47	Improvement
	MeST	*t* (28) = 5.06, *p* < 0.001, *d* = 0.64	Improvement
	Control	*t* (29) = 13.25, *p* < 0.001, *d* = 1.05	Improvement
Follow-up			
Between Group	c-MeST vs Control	*t* (57) = 0.01, *ns*	c-MeST = Control
	MeST vs Control	*t* (57) = 0.01, *ns*	MeST = Control
	c-MeST vs MeST	*t* (56) < 0.001, *ns*	c-MeST = MeST
Within Group	c-MeST	*t* (28) = 8.33, *p* < 0.001, *d* = 0.32	Improvement
	MeST	*t* (28) = 11.87, *p* < 0.001, *d* = 0.79	Improvement
	Control	*t* (29) = 4.32, *p* < 0.001, *d* = 0.25	Improvement

^a^Not significant when Bonferroni correction applied.

#### Emotion regulation

The group × time interactions were significant for adaptive emotion regulation, *F* (3.573, 151.862) = 329.83, *p* < 0.001, η^2^ = 0.88, and maladaptive cognitive emotion regulation, *F* (4, 170) = 264.25, *p* < 0.001, η^2^ = 0.86. As shown in Table 2, compared to the Control group, both the c-MeST and MeST groups had significant improvements in adaptive and maladaptive emotion regulation at post-intervention and follow-up. The c-MeST and MeST groups did not differ significantly at post-intervention or follow-up. While the MeST and c-MeST groups had significant improvements in adaptive emotion regulation (post-intervention and follow-up) and maladaptive emotion regulation (post-intervention only), when compared to baseline, the Controls showed no significant improvements. All three groups showed significant improvements in maladaptive emotion regulation at follow-up, when compared to baseline.

#### Cognitive control

##### Wisconsin card sorting test

The group × time interactions were significant for number of perseverative errors,* F* (2.95, 125.366) = 77.21, *p* < 0.05, *η*^2^ = 0.64, and non-perseverative errors, *F* (3.415, 145.129) = 101.62, *p* < 0.05, *η*^2^ = 0.70. As shown in Table 2, the c-MeST and MeST groups made significantly fewer preservative and non-perseverative errors than Controls at post-intervention and follow-up. The MeST group had fewer perseverative and non-perseverative errors that the c-MeST group at post-intervention and follow-up. When compared to baseline, all groups had significantly fewer errors at post-intervention and follow-up.

##### Stroop test

The group × time interactions were significant for interference error, *F* (3.37, 143.22) = 39.41, *p* < 0.05, *η*^2^ = 0.48, and interference time, *F* (2.59, 110.11) = 4.02, *p* < 0.05, *η*^2^ = 0.08. As shown in Table 2, the c-MeST and MeST groups had fewer errors than Controls at post-intervention and follow-up. The MeST and c-MeST groups did not differ significantly at post-intervention or follow-up. When compared to baseline, only the MeSt and c-MEST groups had significantly fewer errors at post-intervention, but all three groups had fewer errors at follow-up. The three groups did not differ significantly in interference time at post-intervention or follow-up and all groups had improved interference times at post-intervention and follow-up.

#### Mediation analyses

Our analyses showed that changes in memory specificity, B = 1.08*, SE* = 0.40*,* 95% CI [0.18–1.85], adaptive emotion regulation, B = 2.42, *SE* = 0.61*,* 95% CI [1.36–3.71], maladaptive emotion regulation, B = 2.39*, SE* = 0.69*,* 95% CI [1.09–3.83], and aspects of cognitive control (perseverative errors, B = 1.44*, SE* = 0.54, 95% CI [0.49–2.63], non-perseverative errors, B = − 0.68, *SE* = 0.29*,* 95% CI [− 1.35 to − 0.18]), mediated the association between group and follow-up depression scores. There was no evidence that changes in interference error, B = 0.33*, SE* = 0.34, 95% CI [− 0.04 to 1.23], or interference time, B = 0.07, *SE* = 0.26, 95% CI [− 0.03 to 0.94], mediated this association. See supplementary material for figures and further details about the mediation models.

## Discussion

The primary objective of this study was to compare the efficacy of in-person MeST and c-MeST in improving depression among adolescents in Iran. Both in-person MeST and c-MeST were successful in reducing depression symptomatology, when compared to Controls, at post-intervention and follow-up. However, while the c-MeST group and MeST group did not differ significantly at post-intervention, the c-MeST group had significantly greater depression symptomatology than the MeST group at follow-up. Within-group analyses showed that both the MeST group and c-MeST group had significantly lower levels of depression symptoms at post-intervention and follow-up, when compared to baseline. The Control group showed no significant changes in depression. The findings support previous research demonstrating training approaches targeting memory specificity can improve memory retrieval and reduce depression symptoms in adolescents (e.g.,^19,30^). While both c-MeST and MeST seem to have efficacy in reducing depression, MeST may be slightly superior, which may reflect participants in traditional MeST interacting with a psychologist and thus, may feel a greater sense of support. However, if there are resource constraints or clients prefer an on-line version, c-MeST appears to also be beneficial in reducing depression.

Regarding the secondary objectives, c-MeST and MeST improved memory specificity. However, participants in the in-person MeST group showed significantly greater retrieval of specific memories at post-intervention and follow-up compared to the c-MeST group. These findings align with previous studies examining c-MeST^21,22^ and MeST^19^, which showed that memory specificity can be enhanced among depressed individuals using both modes of memory specificity training. However, greater levels of improvement appear to be associated with in-person delivery of MeST. Moreover, we found that improvements in memory specificity mediated improvements in depression. This aligns with previous research that has also demonstrated that changes in memory specificity mediated the relationship between receipt of MeST and reduction in later depression among adolescents^19^. These findings highlight that targeting memory specificity can lead to improvements in depression among adolescents.

The encouraging outcomes of the MeST and c-MeST may reflect reconsolidation mechanisms, involving the refinement of overgeneral memories through the integration of specific information^31^. In addition, as individuals engage in recalling memories encompassing a variety of personal content, there is a reevaluation of one's beliefs about oneself (e.g., self-schema) or one's life, as well as broader concepts like the sense of meaning in life or the development of a more cohesive narrative identity^32^. This is promising given that MeST and c-MeST are both low resource and low-intensity interventions and thus may have utility for adolescents in Iran, particularly as c-MeST can be delivered without the need for facilitators and is self-directed by adolescents.

A secondary objective was to examine whether both in-person MeST and c-MeST could enhance emotion regulation and cognitive control in adolescents. Participants in both groups showed higher levels of cognitive control and emotion regulation following training. Moreover, improvements in both emotion regulation and aspects of cognitive control mediated improvements in depression. It has been suggested that the diminished specificity in recalling positive memories might be intrinsically linked to deficits in cognitive control and the heightened and prolonged adoption of ineffective emotion regulation techniques^23^. The prolonged reliance on these ineffective strategies is likely to hinder the capacity to effectively engage with beneficial mood-regulating strategies, like cognitive reappraisal. Consequently, this may result in the formation of non-specific positive memories, primarily employed to adaptively alleviate negative affective states in most instances^33^. Furthermore, maladaptive autobiographical memory processes typically manifest as the overgeneralized or categorical retrieval of memories. This pattern has been emphasized for its notable positive correlation with mood-congruent memory and emotion regulation in various psychopathological conditions, such as depression^34^. Therefore, it can be concluded that in the present study, as a result of targeting autobiographical memory specificity, emotion regulation and cognitive control improved among participants, and improvements in these domains were associated with improvements in depression symptomatology.

Regarding the limitations of this study, it is important to note that participants were exclusively male. Therefore, there is a need to conduct further research to explore these findings among females as well. Second, since the duration of follow-up for therapeutic effects was limited to 1 month, it seems necessary to conduct a long-term follow-up to assess the sustained therapeutic effects. Third, given the sample size it was not possible to analyze the types of memories being retrieved in the interventions and on the AMT. For example, participants may be recalling trivial memories (e.g., completing my homework last Tuesday) or significant memories (e.g., birth of a sibling). This could be considered in further studies given the role autobiographical memory plays in identity, social interactions, problem-solving and emotion regulation^35^. Fourth, when we applied Bonferroni correction to our post-hoc analyses all the significant findings remained, with the exception of the c-MeST and MeST differences in depression (follow-up), memory specificity and cognitive control no longer being significant. We decided to report these differences given the effect sizes and for transparency but further research is needed to examine the robustness of differences for the two interventions. Fifth, the control group was an inactive control group. Thus, the findings may have been impacted by the interactions, attention and communication of participants with the experimenters in the intervention groups. This should be considered when interpreting findings. Sixth, to align with past c-MeST studies^22^, participants completed c-MEST sessions at their convenience within a 2-week timeframe, while MeST was delivered over a 1-month period. This may have accounted for differences in findings between the two interventions. Finally, we did not assess the socio-economic status of participants, which may have impacted our findings and the generalizability of our findings, and measures of socio-economic status should be included in future studies.

In sum, MeST and c-MeST improved symptoms of depression at post-intervention and these improvements were maintained at 1-month follow-up. Additionally, MeST and c-MeST were found to improve memory specificity, emotion regulation and cognitive control. These initial findings are promising and indicate that MeST and c-MeST may have utility for adolescents in Iran. MeST appears to be slightly superior but if there are resource constraints or clients prefer an on-line version, c-MeST appears to also be beneficial in reducing depression.

## Method

### Participants

Ninety male adolescents between the ages of 13 and 18 years (*M*_*age*_ = 16.36, *SD* = 1.01) who had been recently diagnosed with depression participated in the study. We recruited boys as we could more readily access male students, and the rate of suicide among boys in this region is higher than for girls^13^. Participants were recruited from schools in Mashhad, Iran. Study inclusion criteria were (a) being between 13 to 18 years old, (b) meeting criteria for a diagnosis of major depression according to the Farsi version of the Structured Clinical Interview for DSM-5 (SCID-5-RV^36^), (c) having completed the sixth grade, and (d) having access to the internet. Exclusion criteria were (a) alcohol or other drug abuse or dependence, (b) learning disability, (c) current psychotic disorder, (d) a suicide attempt within the last 6 months, or (e) currently engaging in another psychological treatment. Each participant was screened for depression symptoms using the Beck Depression Inventory (BDI-II)^37^. Participants who scored above the BDI-II clinical cut-off^38^ were invited to attend a clinical interview, whereby the 90 participants met diagnostic criteria for major depression.

### Measures

#### Primary objective measure

##### Beck Depression Inventory (BDI-II)

The BDI-II, consisting of 21 self-report items, measured symptoms of depression^37^. We used the Persian version of the BDI-II, translated and validated by Ghasemzadeh et al.^38^. Total scores on the BDI-II ranged from 0 to 63. Scores falling in the range of 0–9 are considered ‘normal’, 10–16 indicate ‘mild’ depression, 17–20 signify ‘moderate’ depression, 21–30 represent ‘severe’ depression, and scores above 30 suggest ‘very severe’ depression’^38^. The Persian version of the BDI-II demonstrates satisfactory internal consistency (Cronbach’s alpha = 0.87) and test–retest reliability (*r* = 0.74)^38^.

#### Secondary objective measures

##### Autobiographical Memory Test (AMT)

The AMT serves as a gold-standard instrument for assessing OGM (Williams & Broadbent, 1986). Participants were presented negative (5 words) and positive (5 words) cue words, as used in Williams and Broadbent’s^39^ study (we also piloted these words on 10 participants to ensure valence). Participants were tasked with retrieving a specific memory associated with each cue word^39^. Participants received information that they would be provided with ten cue words and their task was to recall and document a specific memory (recent, distant, significant, or interesting) for each cue word. It was emphasized that the events recalled should have a brief duration, lasting less than a day, and occur at a distinct place and time. The importance of specificity in recalling memories was underscored, with illustrative examples provided. Upon participant’s full comprehension of the task, the initiation of the task commenced, allowing thirty seconds for each cue word. If the initially provided memory lacked specificity, participants were encouraged to offer a more specific memory. Following the approach of previous studies (e.g.,^19^), scoring was based on participants generating a specific memory with a duration of less than a day, occurring at a specific time and place (scored as one), or a more general memory (scored as zero). Higher total scores indicated greater autobiographical memory specificity, while lower scores suggested more OGMs. Two assessors, blinded to the study's objectives and the participants group allocation, independently rated all memories. Interrater reliability for specificity and non-specificity was deemed good (k = 0.92).

##### Cognitive Emotion Regulation Questionnaire (CERQ)

The CERQ is a comprehensive questionnaire designed to identify the cognitive emotion regulation strategies employed by an individual following negative events or situations^40^. The short form of the CERQ was developed based on both theoretical and empirical foundations and the 18-item self-report questionnaire assessed nine distinct cognitive emotion regulation strategies. Examples of items “I think that I have to accept that this has happened” (acceptance) and “I often think that I am the cause of what happened” (self-blame). In the current study, we calculated total scores for the adaptive and maladaptive strategies. The CERQ facilitates the identification of individual cognitive strategies^40^. The CERQ-P has undergone assessment in Iranian populations and the scale's validity was established using internal consistency methods, demonstrating Cronbach's alpha values ranging from 0.76 to 0.92 and retest correlations spanning from 0.51 to 0.77^41^.

##### Wisconsin Card Sorting Test (WCST)

The WCST is recognized as a measure of cognitive flexibility and attentional set shifting and is used to evaluate abstraction and the capacity to adapt cognitive strategies to changing environmental conditions^42,43^. The WCST has been used in other studies relating to depression (e.g.,^44^). The WCST involves four stimulus cards displaying a red triangle, two green stars, three yellow crosses, and four blue circles. In the current study the non-computerized version was used. Participants were provided with two sets of 64 response cards, categorized by color, shape, and number. Their task was to match each response card to one of the four stimulus cards, receiving feedback on correctness after each trial. The test assessed the establishment and maintenance of cognitive sets, contributing to its ability to gauge abstraction. Performance indices include the number of achieved categories, perseverative errors (failure to shift cognitive set after negative feedback), and non-perseverative errors (failure to maintain cognitive set after positive feedback)^45^.

##### Stroop Color and Word Test (SCWT)

The SCWT is a widely employed neuropsychological test with applications in both experimental and clinical settings^46^. It was used to evaluate the capacity to inhibit cognitive interference, where the processing of one stimulus feature interferes with the simultaneous processing of another attribute of the same stimulus^46^. Participants were instructed to complete three different tasks as quickly as possible. Two tasks involved reading color words printed in black ink (referred to as the “Fcongruent condition”) and naming color patches (referred to as the “congruent condition”). In contrast, the third task involved reading color words printed in ink colors that did not match the word (referred to as the “incongruent condition”). In this incongruent condition, participants were required to name the ink color instead of reading the word (Reading an isolated word is generally considered a prototypical example of an automatic process. In contrast, color naming tends to be categorized as a controlled process because it is slower, depends on processing strategy, and requires cognitive resources. The basic idea is that automatic processes, such as reading words, cannot be easily suppressed and therefore interferes with controlled processes, such as naming colors.)^47^. In scoring the Stroop task, the interference time for each stage and the number of errors for each participant are recorded. Based on these data, the time differences between different stages, including the word-color stage and other stages, were calculated. These scored reflected the individual's cognitive performance in dealing with various tasks and their skill in coping with cognitive interference.

### Interventions

#### Memory Specificity Training (MeST)

MeST was designed to enhance memory specificity and alleviate OGM^20^. MeST involved six sessions individually delivered by a trained psychologist over a month. The primary focus of MeST was to enhance the precision of retrieving specific personal events from autobiographical memory. The first session introduced the function of memory, with participants practicing recalling specific memories in response to neutral and positive cue words. Homework involved generating memories for five positive and five neutral cue words and a specific memory for each day. Subsequent sessions (2 and 3) reviewed and expanded on the homework, and Session 3 included addressing negative cue words. Sessions 4 and 5 continued this pattern, incorporating negative cue words in homework. These sessions also involved reviewing and practicing recalling specific memories in response to valence cue words. The final session (6) provided additional practice opportunities, guidance on transitioning from specific to unspecific thinking, and a comprehensive summary of the MeST intervention.

#### Computerized Memory Specificity Training (c-MeST)

c-MeST consisted of six sessions that drew upon established techniques for enhancing memory specificity, both in face-to-face settings, as outlined by Raes et al.^20^, and online formats, as described by Takano et al.^22^. Participants practiced generating specific autobiographical memories in response to various cue words during each session. They received feedback on the specificity of these memories through an algorithm designed to assess written memories^22^. Aligning with Takano et al.^22^, participants completed the sessions at their convenience within a 2-week timeframe, with a recommendation to avoid multiple sessions in a single day and to space out the intervention. Although direct monitoring of adherence to this advice was not conducted, the 2-week duration was chosen to enhance participant motivation, considering the success observed in a previous study aimed at improving memory specificity within a similar timeframe^22^. The program was not designed for optimal use on mobile phones, and participants were advised to complete it using a laptop or personal computer.

### Procedure

The study protocol was registered with the Kharazmi University Review Board. The study was approved by the Biomedical Research of Kharazmi University Review Board and Ethics Committee (IR.KHU.REC. 1402.028). The research was performed in accordance with relevant guidelines/regulations and in accordance with the Declaration of Helsinki. Upon obtaining informed consent from adolescents and their parents/guardians, participants underwent individual assessments at three distinct time points: baseline (pre-intervention), post-intervention (after the intervention), and 1-month post-intervention (follow-up). Following the baseline assessment participants were randomly allocated using computer-generated randomization to three groups: MeST group (*n* = 30), the c-MeST group (*n* = 30) and the non-active control group (*n* = 30). Every assessment included the BDI-II, AMT, CERQ, WCST and SCWT. The assessments were conducted by a researcher blinded to group assignment.

### Data analysis plan

There was no missing data for all participants included in the analyses. Following the approach of previous MeST studies (e.g.,^19,48^), we conducted a series of 3 (MeST, c-MeST, control) × 3 (baseline, post-intervention, follow-up) mixed-design analyses of variances (ANOVAs), with (a) depression symptoms, (b) memory specificity (AMT scores), (c) adaptive emotion regulation (CERQ scores), (d) maladaptive emotion regulation (CERQ scores), (e) WCST scores, and (f) SCWT scores as the dependant variables to examine the effects over time. Eta squared effect sizes were computed for the ANOVA analyses, wherein values of 0.01 were interpreted as small, 0.06 as medium, and 0.14 as large^49^. Subsequent to identifying significant interactions, post-hoc *t*-tests were employed for further exploration, accompanied by the calculation of Cohen d effect sizes (magnitudes of 0.20, 0.50, and 0.80 were categorized as small, moderate, and large, respectively^49^). Finally, we conducted mediation analyses exploring whether changes (i.e., post-intervention scores − baseline scores) in memory specificity, emotion regulation and cognitive control mediated depression symptom improvement at follow-up using PROCESS (Model 4)^50^. Significance was indicated by the 95% confidence interval not including zero. For the memory specificity analyses (AMT scores), we also considered positive and negative words separately and did not find any differences to that reported above for the overall score.

### Ethical approval

The study protocol was approved by the local Ethics Committee (with the approval ID: IR.KHU.REC. 1402. 028) in Biomedical Research of Kharazmi University, Tehran, Iran. The research was performed in accordance with relevant guidelines/regulations and in accordance with the Declaration of Helsinki. Participants and their parents/guardians provided written informed consent.

## Supplementary Information

Supplementary Information.

## Data Availability

The data that support the findings of this study are available from the corresponding author upon reasonable request.
