# Supplementary material for: Exploring the efficacy of memory specificity training on depression among Iranian adolescents: a comparative analysis of online vs. in-person delivery
Source: Sci Rep. 2024 Sep 28;14:22412. doi: 10.1038/s41598-024-68709-9 (PMC11438878; doi:10.1038/s41598-024-68709-9)
Supplement: Supplementary file 1 — Supplementary Information. [file 41598_2024_68709_MOESM1_ESM.docx]

**Supplementary Material – Mediation Analyses**

**Mediator: AMT**

Mediation (AMT)

b

a

Independent variable (Group)

Dependant variable (BDI)

c’

’’

| a pathway | b pathway | c’ pathway |
| --- | --- | --- |
| b = -1.07 | b = -1.00 | b = 6.83 |
| SE = .18 | SE = .48 | SE = .98 |
| p < .01 | p = .03 | p < .001 |

Indirect effect: b = 1.08, SE = .40 The 95% CI [0.18-1.85] does not contain zero

**Mediator: Adaptive cognitive emotion regulation**

Mediation (Adaptive cognitive emotion regulation)

b

a

c’

’’

Independent variable (Group)

Dependant variable (BDI)

| a pathway | b pathway | c’ pathway |
| --- | --- | --- |
| b = -2.61 | b = -.92 | b = 5.48 |
| SE = .46 | SE = .17 | SE= .87 |
| p < .01 | p <.01 | p <.001 |

Indirect effect: b = 2.42, SE = .61. The 95% CI [1.36-3.71] does not contain zero.

**Mediator: Maladaptive cognitive emotion regulation**

Mediation (Maladaptive cognitive emotion regulation)

b

a

Dependant variable (BDI)

Independent variable (Group)

c’

’’

| a pathway | b pathway | c’ pathway |
| --- | --- | --- |
| b = 2.91 | b = .82 | b = 5.52 |
| SE = .39 | SE = .21 | SE = 1.02 |
| p < .01 | p <.01 | p < .01 |

Indirect effect: b = 2.39, SE = 0.69. The 95% CI [1.09-3.83] does not contain zero.

**Mediator: Perseverative errors**

Mediation (perseverative errors)

b

a

c’

’’

Independent variable (Group)

Dependant variable (BDI)

| a pathway | b pathway | c’ pathway |
| --- | --- | --- |
| b = -2.25 | b = .-64 | b = 6.46 |
| SE = .37 | SE = .23 | SE = .98 |
| p < .01 | p <.01 | p <.001 |

Indirect effect: b = 1.44, SE = .54. The 95% CI [0.49-2.63] does not contain zero.

**Mediator: Non-perseverative errors**

Mediation (non-perseverative errors)

b

a

Independent variable (Group)

Dependant variable (BDI)

c’

’’

| a pathway | b pathway | c’ pathway |
| --- | --- | --- |
| b = 1.10 | b = -.62 | b = 8.59 |
| SE = .40 | SE = .22 | SE = .85 |
| p < .01 | p <.01 | p <.001 |

Indirect effect: b = -0.68, SE = .29. The 95% CI [-1.35- -0.18] does not contain zero.

**Mediator: Interference error**

Mediation (interference error)

b

a

c’

’’

Independent variable (Group)

Dependant variable (BDI)

| a pathway | b pathway | c’ pathway |
| --- | --- | --- |
| b = .33 | b = .99 | b = 7.58 |
| SE = .19 | SE = .45 | SE = .84 |
| p =.10 | p =.03 | p <.001 |

Indirect effect: b = 0.33, SE = .34 The 95% CI [-.04 - 1.23] does contain zero.

**Mediator: Interference time**

Mediation (interference time)

b

a

c’

’’

Independent variable (Group)

Dependant variable (BDI)

| a pathway | b pathway | c’ pathway |
| --- | --- | --- |
| b = .59 | b = .12 | b = 7.83 |
| SE = 1.17 | SE = .07 | SE = .84 |
| p = .61 | p =. 10 | p <.001 |

Indirect effect: b = .07, SE = .26. The 95% CI [-0.03 - 0.94] does contain zero.
